# A male-sterile mutant with necrosis-like dark spots on anthers was generated in cotton

**DOI:** 10.3389/fpls.2022.1102196

**Published:** 2023-01-09

**Authors:** Jun Zhang, Peng Wu, Ning Li, Xiaolan Xu, Songxin Wang, Siyuan Chang, Yuping Zhang, Xingxing Wang, Wangshu Liu, Yizan Ma, Hakim Manghwar, Xianlong Zhang, Ling Min, Xiaoping Guo

**Affiliations:** ^1^ National Key Laboratory of Crop Genetic Improvement & Hubei Hongshan Laboratory, Huazhong Agricultural University, Wuhan, China; ^2^ Zhejiang Provincial Key Laboratory of Crop Genetic Resources, Institute of Crop Science, Plant Precision Breeding Academy, College of Agriculture and Biotechnology, Zhejiang University, Hangzhou, China; ^3^ State Key Laboratory for Conservation and Utilization of Subtropical Agro-Bioresources, South China Agricultural University, Guangzhou, China

**Keywords:** cotton, GhEMS1s, CRISPR/Cas9, male-sterile line, necrosis-like dark spots

## Abstract

Although conventional hybrid breeding has paved the way for improving cotton production and other properties, it is undoubtedly time and labor consuming, while the cultivation of male sterile line can fix the problem. Here, we induced male sterile mutants by simultaneously editing three cotton *EXCESS MICROSPOROCYTES1* (*GhEMS1*) genes by CRISPR/Cas9. Notably, the *GhEMS1* genes are homologous to *AtEMS1* genes, which inhibit the production of middle layer and tapetum cells as well, leading to male sterility in cotton. Interestingly, there are necrosis-like dark spots on the surface of the anthers of *GhEMS1s* mutants, which is different from *AtEMS1* mutant whose anther surface is clean and smooth, suggesting that the function of *EMS1* gene has not been uncovered yet. Moreover, we have detected mutations in *GhEMS1* genes from T_0_ to T_3_ mutant plants, which had necrosis-like dark spots as well, indicating that the mutation of the three *GhEMS1* genes could be stably inherited. Dynamic transcriptomes showed plant hormone pathway and anther development genetic network were differential expression in mutant and wild-type anthers. And the lower level of IAA content in the mutant anthers than that in the wild type at four anther developmental stages may be the reason for the male sterility. This study not only facilitates the exploration of the basic research of cotton male sterile lines, but also provides germplasms for accelerating the hybrid breeding in cotton.

## Introduction

Male sterility is an important tool for the utilization of heterosis such as increasing cotton production and quality with less labor and time. For now, artificial emasculation is still the dominant method used for the production of cotton hybrids in China (Yang et al., 2018). However, the cost of hybrid breeding has been increasing year by year due to the shortage of rural labor, resulting in dramatic decreases of production in the planting area of hybrid cotton (Yang et al., 2018; Zheng et al., 2021). In this way, the creation of male sterile lines is a new breakthrough for the acquisition of hybrid seedlings.

Recently, the CRISPR/Cas9 technology has been widely used in gene editing and the plants acquired can be used for hybrid seed production. Due to its precision, simple operation and high efficiency, the CRISPR/Cas9 technology has been applied for a variety of species such as maize, wheat, soybean and rice (Chen et al., 2018; Ma et al., 2019; Okada et al., 2019; Chen et al., 2021). Novel “transgene clean” thermo-sensitive genic male sterility (TGMS) lines have been created on the basis of the induced specific mutations in *TMS5* with the CRISPR/Cas9 technology. To test the combinatorial capacity of the obtained new male sterile mutants, the rice *TMS5* mutants have crossed with other lines and found that the offspring have better phenotypes and provide higher yields (Zhou et al., 2016). In addition, using the CRISPR/Cas9 technology to target *ABORTED MICROSPORES* (*AMS*) congeners in soybeans to produce stable male sterility lines. Furthermore, they have eventually figured out that the editing of *GmAMS1* is related to not only the formation of the pollen wall but also the degradation of the tapetum (Chen et al., 2021). Ramadan et al. have successfully generated a wide scale of genotypically and phenotypically mutagenesis using CRISPR/Cas9 mediated pooled sgRNAs assembly, paving the way for creation of cotton male sterile lines (Ramadan et al., 2021).

As shown in the anther and pollen-related gene regulatory network diagram (Wilson and Zhang, 2009), the early anther cell differentiation gene *EXCESS MICROSPOROCYTES1/EXTRA SPOROGENOUS CELLS* (*EMS1/EXS*) encodes leucine-rich repeat (LRR) receptor kinase which is located on the cell membrane, and the protein is expressed in primary cell wall and tapetum cells. Hence, mutations in *EMS1/EXS* gene is related to the absence of tapetum in *Arabidopsis thaliana*, eventually resulting in pollen abortion (Canales et al., 2002). Moreover, the *MULTIPLE SPOROCYTE* (*OsMSP1*) gene in rice is homologous to the *AtEMS1* genes, which also encodes LRR receptor kinases as well, and the *OsMSP1* mutant exhibits a highly similar phenotypes to the *Arabidopsis EMS1/EXS* mutant (Ken-Ichi Nonomura et al., 2003). Therefore, the *EMS1* is an important candidate gene for obtaining male sterile lines. At the same time, we also found that the expression of *GhEMS1* was affected in the high temperature sensitive line by high temperature. Here, we have created a complete male sterile line by knocking out the *GhEMS1s* using CRISPR/Cas9 technology. Through the comparison of pollen fertility and tapetum development of edited male sterile lines, the most suitable mutant was identified. This study not only facilitates the exploration of the basic research of cotton sterile lines, but also provides germplasms for accelerating the hybrid breeding using male sterile lines in cotton.

## Materials and methods

### Plant materials and growth conditions

Jin668, an upland cotton (*Gossypium hirsutum* L.) line developed by the National Key Laboratory of Crop Genetic Improvement, Huazhong Agricultural University. We have described the transformation system of Jin668 previously (Jin et al., 2006; Li et al., 2019). The wild-type (negative control) and transgenic lines were planted in Wuhan, Hubei under normal farming practices or grown in the greenhouse during the winter in 2018 - 2020. The greenhouses were kept at a temperature of 28–35/20–28°C day/night.

### Vector construction and transformation of cotton

We conducted a genome-wide assessment and chose sgRNA through the CRISPR-P 2.0 (http://crispr.hzau.edu.cn/cgi-bin/CRISPR2/CRISPR) (Liu et al., 2017). The process of vector construction refers to our previous report (Wang et al., 2018). The different vectors were transformed into Agrobacterium *GV3101* which then was transformed into cotton Jin668. Refer to published articles for cotton transgene (Jin et al., 2006).

### Hi-TOM and gene editing efficiency

In order to detect the editing efficiency of transgenic lines, the targeted genomic DNA was amplified by PCR with a pair of site-specific primers at the 5’ end with common bridging sequences. Specific steps were similar to previous reports (Liu et al., 2019; Ramadan et al., 2021), and the primers used ware shown in **Supplementary Table 1**. PCR products were sequenced on Illumina HiSeq platform (Illumina, USA) after recovery. Hi-TOM website (http://www.hi-tom.net/hi-tom/) was used to analyze the sequencing results. In order to detect the off-target situation in the transgene lines editing process, “sgRNAcas9_3.0.5” (Xie et al., 2014) was used to predict off-target sites, following the software default settings. The “extract_targetSeq.pl” script was used in the software package to extract the flanking sequence of the off-target site on the genome. We designed off-target site primers in batches through the “batchprimer3” website (http://batchprimer3.bioinformatics.ucdavis.edu), PCR products were sequenced on Illumina HiSeq platform (Illumina, USA). “CRISPResso2” (Clement et al., 2019) was employed for sequencing results to analyze the off-target sites. The off-target sites and sequences are shown in **Supplementary Table 2**, **3**. The primers for amplification of off-target sites are shown in **Supplementary Table 1**.

### Observation of anther phenotype

To detect pollen viability, the anther at 0 days post-anthesis (DPA) of WT and mutants was immersed in 2,3,5-Triphenyl tetrazolium chloride (TTC) solution (8 g TTC dissolved in 1 L phosphate buffer) according to a previous report (Min et al., 2013). After being cultured in a 37°C incubator for 30 min, the staining reaction was terminated with 2% (v/v) sulfuric acid solution. Pollen grains were placed on a microscope slide and the Zeiss (Oberkochen, Germany) Axio Scope A1 microscope was used to collect images.

### Polyacrylamide gel electrophoresis

In polyacrylamide gel electrophoresis (PAGE) separations, the 8% non-denaturing polyacrylamide gel (acrylamide: methylene bisacrylamide = 29:1) containing the PCR amplification products were placed in the electrophoresis chamber, and the driving force was set to 60 W. After 1 hour, the sample products were immersed in 0.2% silver nitrate solution for 10 minutes. After that, the products were washed twice with ddH_2_O and then put them in the chromogenic solution (1.5% sodium hydroxide, 0.4% formaldehyde) for 5 minutes. Finally, protein band patterns could be visualized and subjected to adequate analysis.

### Tissue dissection and PCD assays

Anthers from transgenic lines and wild-type at different developmental stages were immersed in 50% FAA (50% ethanol, 5% propionic acid, and 3.7% formaldehyde) and vacuum infiltrated for 2 h at 4°C, and placed at 4°C for 24 h to fix the tissue. For dehydration, a graded ethanol series (50, 70, 80, 95, and 100%) was used and samples were embedded in paraffin. The embedded tissues were sectioned into 10 μm sections. Anther sections were stained with toluidine blue solution (1%) and the Zeiss (Oberkochen, Germany) Axio Scope A1 microscope was used to collect images. TUNEL detection of apoptosis was performed similar to previous report (Min et al., 2013). Paraffin sections of the anthers were used for TUNEL analysis of the fragmented DNA of apoptotic cells using the DeadEnd™ Fluorometric TUNEL System (G3250, Promega). The analytical wavelengths of fluorescein and propidium iodide were 520 ± 10 nm and 640 ± 10 nm by a confocal microscope (TCS SP2; Leica), respectively.

### RNA extraction and RNA-seq

Anthers from transgenic lines and wild-type were sampled and total RNA was extracted. The library preparations were sequenced on an Illumina Novaseq platform and 150 bp paired-end reads were generated. Raw data of fastq format were firstly processed through FastQC (Andrianov et al., 2010). Paired-end clean reads were aligned to the *G. hirsutum* genome using Hisat2 v2.0.5 (Kim et al., 2015). FeatureCounts v1.5.0-p3 was used to count the reads numbers mapped to each gene (Liao et al., 2014). And then fragments per kilobase of exon model per million mapped fragments of each gene was calculated based on the length of the gene and reads count mapped to this gene. Differential expression analysis of two groups was performed using the DESeq2 R package (1.16.1) (Love et al., 2014). Genes with Padj <0.05 and |log_2_FoldChange| >1 were assigned as differentially expressed. The GO enrichment was performed by the R package ‘clusterProfiler’ (Yu et al., 2012).

### Hormone determination

Extraction and measurement of the endogenous IAA were as described by Miao et al. (Miao et al., 2019). Three replicates, each of 100 mg of anthers from transgenic lines and wild-type, were sampled at anther developmental stage 6, 7, 9 and 10, mixed with 750 μL of ice-cold 80% methanol containing ^2^H_5_-IAA (OlChemlm Ltd, CAS: 76934-78-5, 10 ng ml^-1^) as internal standard, and shake for 16 hours in the dark at 4°C. After centrifugation at 13,000 rpm for 5 minutes, the supernatant was dried with nitrogen, and the residue was reconstituted in 300 μL of 80% methanol. Finally, the IAA content was measured using an Agilent 4000Q-TRAP HPLC-MS system.

## Results

### Identification of EMS1 genes in *G. hirsutum*

**Figure 1:**
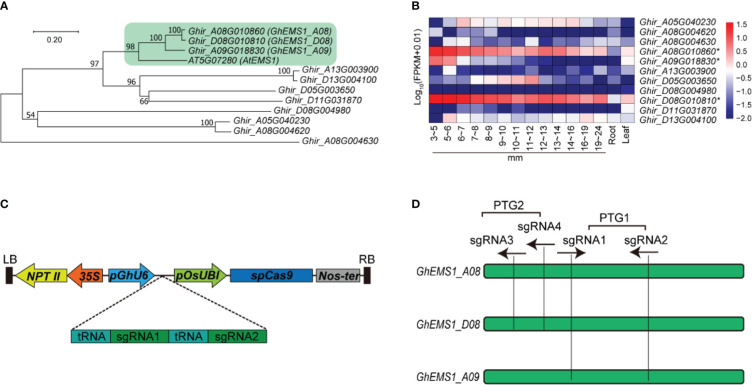
Creation of *Gh*EMS1 genes mutants by using CRISPR/Cas9. **(A)** A phylogenetic tree for EMS1 genes in *Gossypium hirsutum* and AtEMS1 was constructed using the neighbor-joining method in MEGA 10.1.8 followed by bootstrapping with 1,000 replicates; **(B)** Expression profiles of *GhEMS1* genes in flower buds of different lengths (3~5 mm, 5~6 mm, 6~7 mm, 7~8 mm, 8~9 mm, 9~10 mm, 10~11 mm, 11~12 mm, 12~13 mm, 13~14 mm, 14~16 mm, 16~19 mm, 19~24 mm)in *G. hirsutum* H05 determined by transcriptome sequencing (Zhang et al., 2022); **(C)** Creation of a male-sterile line pool by CRISPR/Cas9. Two sgRNAs were serially connected to each vector to increase the knockout efficiency; **(D)** Two pairs of sgRNAs on exons were designed and vectors containing polycistronic tRNA–gRNA genes (*PTG*). *Ghir_A08G010860 (GhEMS_A08), Ghir_D08G010810 (GhEMS_D08)*, and *Ghir_A09G018830 (GhEMS_A09)* were knocked out by sgRNA1 and sgRNA2 (*PTG1*). *GhEMS_A08* and *GhEMS_D08 (PTG2)* were knocked out by sgRNA3 and sgRNA4 (*PTG2*).

The *Arabidopsis EXCESS MICROSPOROCYTES1* (*AT5G07280*, *AtEMS1*) controls somatic and reproductive cell development in anthers (Zhao et al., 2002). To determine whether the *EMS1* gene participated in the reproductive cell development in cotton, we used *AtEMS1* as a query to perform BLASTP searches and identified 11 *EMS1* members in *G. hirsutum*. To get a better understanding of the phylogenetic relationships between *EMS1*, a phylogenetic tree was constructed based on these 11 *G. hirsutum EMS1* and *AtEMS1* protein sequences. Clearly, the EMS1s were classified into four branches, the *Ghir_A08G010860* (*GhEMS1_A08*), *Ghir_D08G010810* (*GhEMS1_D08*), and *Ghir_A09G018830* (*GhEMS1_A09*) in the same branch with *AtEMS1* (**Figure 1A**). *GhEMS1_A08*, *GhEMS1_D08* and *GhEMS1_A09* have leucine rich repeat N-terminal domain and leucine-rich repeat sequences (**Supplementary Figure 1**). This result indicated that the three *GhEMS1* genes may have similar functions with *AtEMS1*.

To further identify the biological function of the *EMS1* genes involved in cotton-specific developmental processes, we have summarized the expression of *EMS1* genes in different organs/tissues (including roots, leaves, anthers in different length buds) of *G. hirsutum*. As shown in **Figure 1B**, most of *GhEMS1* genes have expressed in anthers, and *Ghir_A08G010860* (*GhEMS1_A08*), *Ghir_D08G010810* (*GhEMS1_D08*), and *Ghir_A09G018830* (*GhEMS1_A09*) were all predominantly expressed in early-stage anthers (stage 4/5, bud length: 3~5 mm) and their expression gradually decreased with anthers development, implying that these genes may play crucial roles in identification of reproductive cell development in early stage anthers.

### Knockout of *Gh*EMS1 genes using CRISPR/Cas9 caused male sterility with necrosis-like dark spots on the anther surface

**Figure 2:**
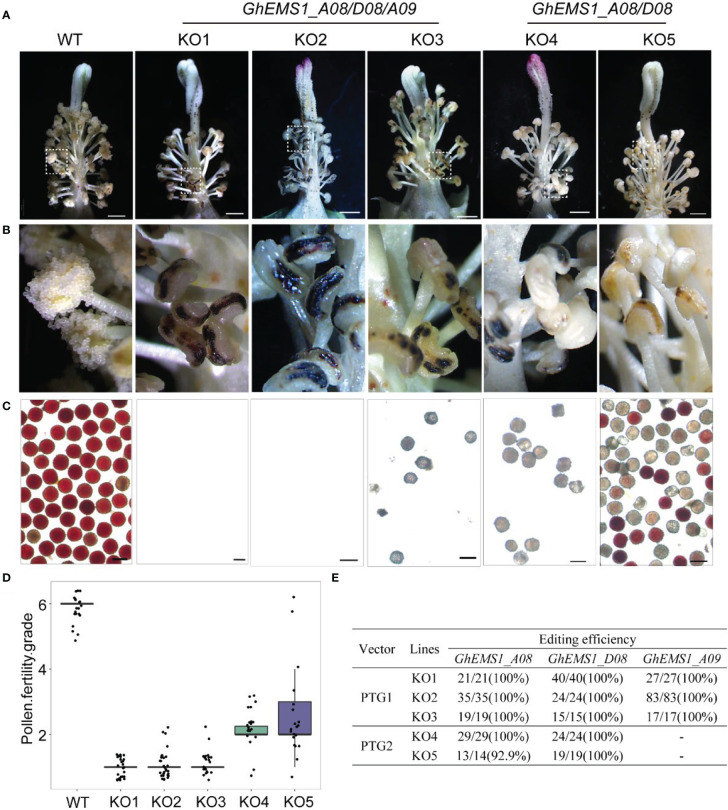
Phenotypes of *GhEMS1s* mutants. **(A)** The phenotypes of the WT and KO1 - KO5 transgenic lines, three genes *Ghir_A08G010860* (*GhEMS_A08*), *Ghir_D08G010810* (*GhEMS_D08*), and *Ghir_A09G018830* (*GhEMS_A09*) of *PTG1* in KO1- KO3 sterile plants. *GhEMS_A08* and *GhEMS_D08* (*PTG2*) were knocked out in KO4 and KO5 plants. Scale bars: 2 mm; **(B)** Partial enlarged view of anther in picture (a); **(C)** TTC (2,3,5-triphenyl tetrazolium chloride) was used to detect the pollen viability. No pollen was observed in the anthers of KO1 and KO2 plants. Scale bars: 100 µm. **(D)** Statistics for pollen fertility in the *GhEMS1* mutants. During the phenotypic investigation, we divided plant fertility into six levels: Grade 1 indicates that anthers with necrosis-like dark spots and all anthers without pollen; Grade 2 indicates that < 25% of the anthers have a few inactive pollen grains without dehiscence; Grade 3, 4, and 5 indicate that 25%, 50%, and 75% of the anthers spread pollen, respectively; Grade 6 indicates that all anthers dehiscence and release active pollen. The phenotype of every flower was recorded, and the fertility of different plants was counted. **(E)** Analysis of gene editing efficiency in different *GhEMS* mutant lines by Sanger sequencing.

Due to combination of two sgRNAs with tRNA can improve the transcription and knockout efficiency (Xie et al., 2015; Wang et al., 2018). Thus, two sgRNAs targeting the same *GhEMS1* genes were combined by overlap extension PCR and then ligated to the expression vector pRGEB32-GhU6.7, to produce polycistronic tRNA-gRNA genes *PTG1* and *PTG2* vectors (**Figure 1C**). The *PTG1* contained sgRNA1 and sgRNA2, which was designed to knock out *GhEMS1_A08*, *GhEMS1_D08*, and *GhEMS1_A09* (**Figure 1D**). The *PTG2* contained sgRNA3 and sgRNA4, which was designed to knock out *GhEMS1_A08* and *GhEMS1_D08* at the same time (**Figure 1D**). The prepared vectors were transformed the cotton by Agrobacterium (*GV3101*) (**Supplementary Figure 2**). We obtained 3 sterile plants (KO1~3) for *PTG1*, and 2 sterile plants (KO4~5) for *PTG2* (**Figure 2A**). Five independent transformation plants showed different degrees of necrosis-like dark spots on the surface of anther and have different pollen viability (**Figures 2A-C**). KO1-KO3, showed obvious necrosis-like dark spots on the surface of the anthers (**Figures 2A, B**). There was no pollen in the anthers of KO1 and KO2, and only a few shriveled pollen grains in the KO3 anthers (**Figure 2C**). KO4 and KO5 showed a few anthers with dark spots, and the pollen quantity and viability were lower than the wild type (WT) but higher than those of KO1- KO3 (**Figures 2A-C**).

Furthermore, a three-month fertility assay was performed on WT and the five transgenic plants. We divided plant fertility into six levels: Grade 1 indicates that anthers with necrosis-like dark spots and all anthers without pollen; Grade 2 indicates that < 25% of the anthers have a few inactive pollen grains without dehiscence with a few anthers have dark spots; Grade 3, 4, and 5 indicate that 25%, 50%, and 75% of the anthers spread pollen, respectively; Grade 6 indicates that all anthers dehiscence and release active pollen. The phenotype of every flower was recorded, and the fertility of different plants was counted. The results showed that the fertility of the WT plants was relatively stable at grade 6, with pollen viability higher than 99.5% and anthers normal dehiscence and no dark spots; KO1- KO3 were below grade 2, with no pollen or very few inactive pollen grains, and dark spots on the anther surface (**Figures 2C, D**). KO1 was the most stable, with 100% anthers of all flowers having necrosis-like dark spots on the anther surface, and no pollen (**Figure 2D**). The fertility of KO4 and KO5 was above grade 2 and below grade 6, with a few anthers have dark spots (**Figures 2B-D**).

### Identification of target gene editing in male sterile plants

To check the mutation at the selected target site in KO1-KO5 lines, the sanger sequencing was performed. We found that three genes, *GhEMS1_A08*, *GhEMS1_D08* and *GhEMS1_A09*, all were 100% edited in KO1-KO3 plants (**Figure 2E**), and the deletion length was in the range of 2 to 29 bp (**Supplementary Figure S3**). In the KO4 and KO5 plants, *GhEMS1_A08* and *GhEMS1_D08* were successfully edited with 100% editing efficiency, and no editing in the *GhEMS1_A09* (**Figure 2E** and **Supplementary Figure S3B**). Moreover, the expression level of *GhEMS1_A09* was lower in the early stage anthers, compared with the expression of *GhEMS1_A08* and *GhEMS1_D08* in the same stage anthers, and the expression level of *GhEMS1_A09* decreased earlier (**Figure 1B**). In all, the male fertility of KO4 and KO5 was better than that of KO1-KO3 (**Figure 2C**), indicating that the three *GhEMS1* genes were essential for male fertility and played crucial roles in male fertility. In addition, 38 potential off-target genes of the two sgRNAs in KO1 (complete male sterility plant) were analyzed, and no off-target effects were found in KO1 (**Supplementary Tables 2**, **3**), this result suggested the formation of male sterility of KO1 only caused by the mutations of *GhEMS1_A08*, *GhEMS1_D08* and *GhEMS1_A09* genes.

### *GhEMS1* mutants displayed the genetic stability of the necrosis-like dark spots as a marker of male sterility

**Figure 3:**
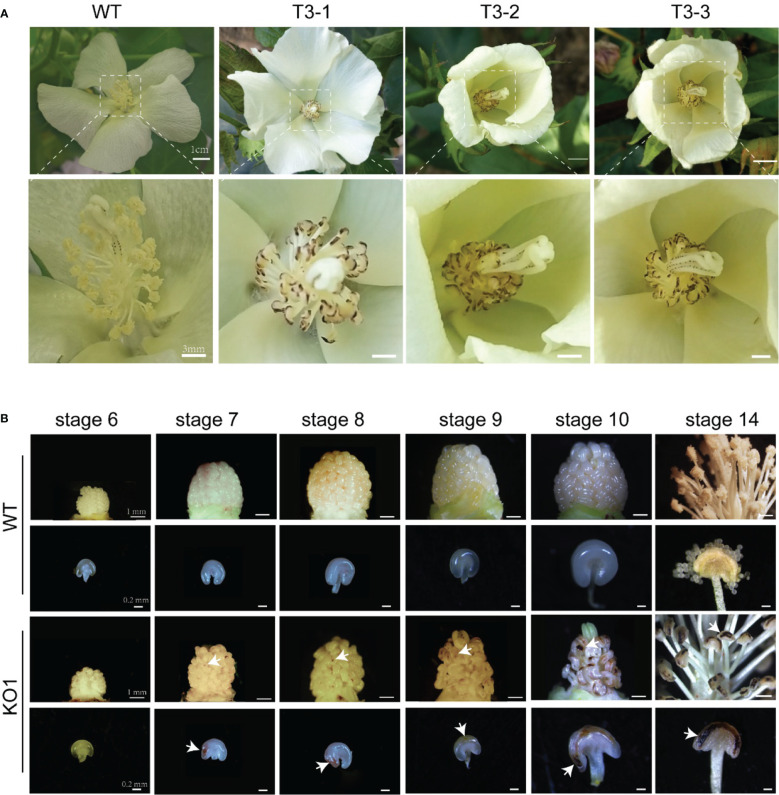
The sterile phenotype of surface necrosis of T3. **(A)** wild type, KO1 T3 plants, and enlarged images. **(B)** Changes in the anthers with different degrees of necrosis-like spots. Compared with those of the WT, yellow spots appeared on the anthers of sterile KO1 plants at stage 7, these spots gradually deepened at stage 10 and stage 14 to form necrosis-like dark spots eventually. KO1, *GhEMS* 3-genes simultaneous mutant. The white arrows indicate necrotic spots.

**Figure 4:**
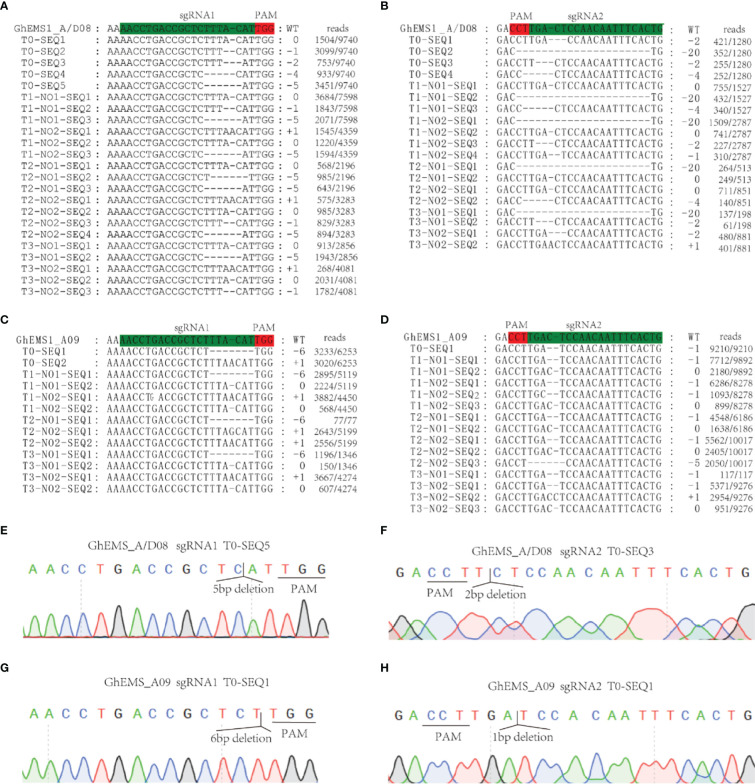
KO1 editing types were inherited in T0 to T3. **(A, B)** *GhEMS_A/D08* were edited in sgRNA1 and sgRNA2, respectively. **(C, D)** *GhEMS_A09* were edited in sgRNA1 and sgRNA2, respectively. **(E)** Sequencing peak photos of *GhEMS_A/D08* with 5 bp deletions at the sgRNA1 site. **(F)** Sequencing peak photos of *GhEMS_A/D08* with 2 bp deletions at the sgRNA2 site. **(G)** Sequencing peak photos of *GhEMS_A09* with 6 bp deletions at the sgRNA1 site. **(H)** Sequencing peak photos of *GhEMS_A09* with 1 bp deletions at the sgRNA2 site.

Whether the sterile phenotype can be stably inherited to the offspring is related to the successful application of sterile mutant to cross breeding. To test the genetic stability of the necrosis-like dark spots as a marker of sterility, we applied WT pollen to the stigma of sterile KO1 plants. Then, the target site fragments in *PTG1* of *GhEMS1_A08*, *GhEMS1_D08* and *GhEMS1_A09* in WT, KO1, T1 generation (KO1×WT) were amplified by PCR, and polyacrylamide gel electrophoresis (PAGE) was used to identify the editing. The results showed that the T1 generation had the same fragment with T0 generation, but due to WT pollination, some new editing types were generated, such as in T1-3, for which two sgRNAs generated new editing types (**Supplementary Figure 3C**). The individual sgRNAs showed different efficiencies in the detection of PAGE, indicating that it is necessary to connect two sgRNAs in tandem with one vector (**Supplementary Figure 3C**). In addition, the necrosis-like dark spots on the anthers could also be observed from the T0 to T3 (**Figures 2A, B** and **3A**), T3 plants (n=64) had 35.9% (23/64) complete necrosis anthers (**Figure 3A**). Gene editing types were identified by Hi-TOM (Liu et al., 2019), which revealed that the T3 generation had the same editing type as the T0 generation, such as the mutations (-1 bp, -5 bp, -6 bp) at the sgRNA1 target site (**Figures 4A, C, E, G**), and mutations (-2 bp, -20 bp, -1 bp) at the sgRNA2 target site (**Figures 4B, D, F, H**). However, due to WT pollination and the retention of Cas9, some new editing types have also been generated, such as the mutation (+1 bp) at the sgRNA1 target site (**Figure 4A**). Moreover, the pollen quantity and viability of T3 plants were analyzed, the results were consistent with the gene editing efficiency and the number of necrosis-like dark spots on the anthers in these plants, suggested the *GhEMS1* mutants displayed the genetic stability of the necrosis-like dark spots as a marker of male sterility.

### *GhEMS1s* mutants lack of middle and tapetum layers might cause delayed microsporocytes development

The necrosis-like dark spots on the mature anthers could be inherited, but when and how the necrosis-like dark spots appeared on the anther surface? To explore the formation period of necrosis-like dark spots, we obtained the stage 6 to stage 14 anthers of *KO1*. At stage 7, yellow spots appeared on the anthers of sterile phenotype plants, which gradually deepened at stage 10 and stage 14 to form necrosis-like dark spots eventually (**Figure 3B**). Therefore, the dark spots on the surface of the anthers started earlier, which can help us to screen sterile plants at the early stage.

**Figure 5:**
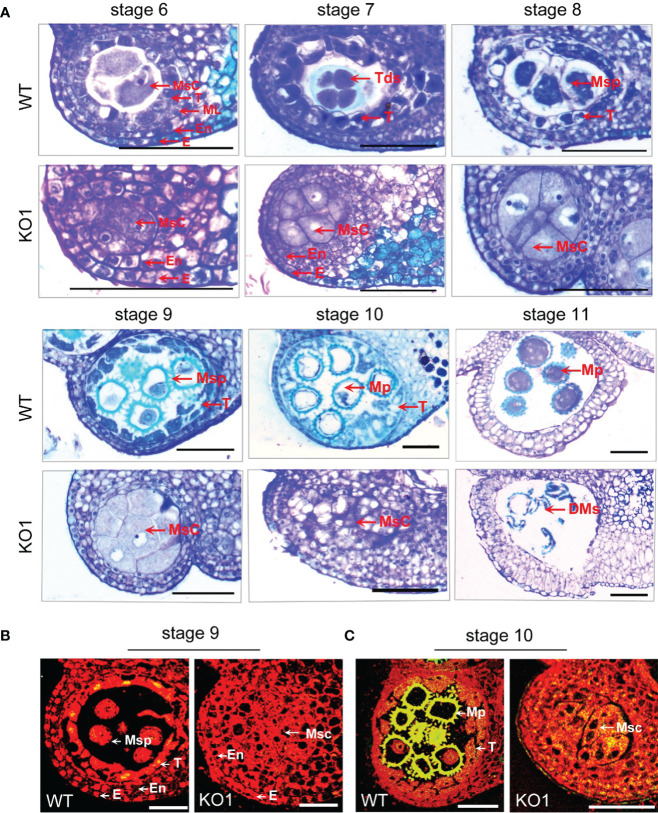
Comparison of the histological characteristics of the WT and KO1 sterile line T3 anthers. **(A)** Stage 6-11 histological characteristics of the WT and KO1, Scale bars: 100 µm **(B, C)** Analysis of DNA damage in anthers of WT and KO1 male-sterile plants. The degree of DNA fragmentation of anthers was detected by TUNEL. Scale bars: 100 µm. DMs: degenerated microspores; E, epidermis; En, endothecium; Msc, microsporocyte; Mp, mature pollen; ML, middle layer; Msp, microspore; T, tapetum; Tds, tetrads; WT, wild type.

To observe the microspore development of *KO1*, anther tissue cross-sections were made. At stage 6, the WT exhibited four complete anther cell layers (from outside to inside: epidermis, endothecium, middle layer, and tapetum layer) and microsporocytes. However, the sterile mutant anthers lacked middle layer and tapetum cells (**Figures 5A, C**). Furthermore, in the WT anthers, the microsporocytes completed nuclear division at stage 6 (**Figure 5A**), tetrads formed at stage 7 (**Figure 5A**), microspores were released from tetrads at stage 8 (**Figure 5A**), and then microspores developed into mature pollen during stage 9 to stage 11 with the tapetum development and degeneration (**Figure 5A**). However, the mutants could complete nuclear division but not cytoplasmic division, causing the enlarged microsporocytes and undetached microsporocytes at stages 7 to 9 (**Figure 5A**). At stage 10, the microsporocytes of mutants started to degrade, and they were completely degraded at stage 11 (**Figure 5A**). What’s more, the degree of DNA fragmentation in the anthers was detected by TUNEL (terminal deoxynucleotidyl transferase dUTP nick end labeling). In the WT, there were a few yellow fluorescence signals in the tapetum layer, a few microspores at stage 9, and the yellow fluorescence signals were observed enhanced at stage 10. However, no fluorescence signals were observed in the stage 9 anthers of the mutant, and only faint fluorescence signals were observed appearing in the degrading microsporocytes in the stage 10 anther locules in the mutant (**Figures 5B, C**). This result indicated that there was no normally developed pollen in the chamber of the mutant and pollen deformity was caused by the absence of tapetum formation and the PCD process of microspore mother cells.

### Dynamic transcriptomes analysis between *Ghems1s* male sterile mutants and wild-type anthers

**Figure 6:**
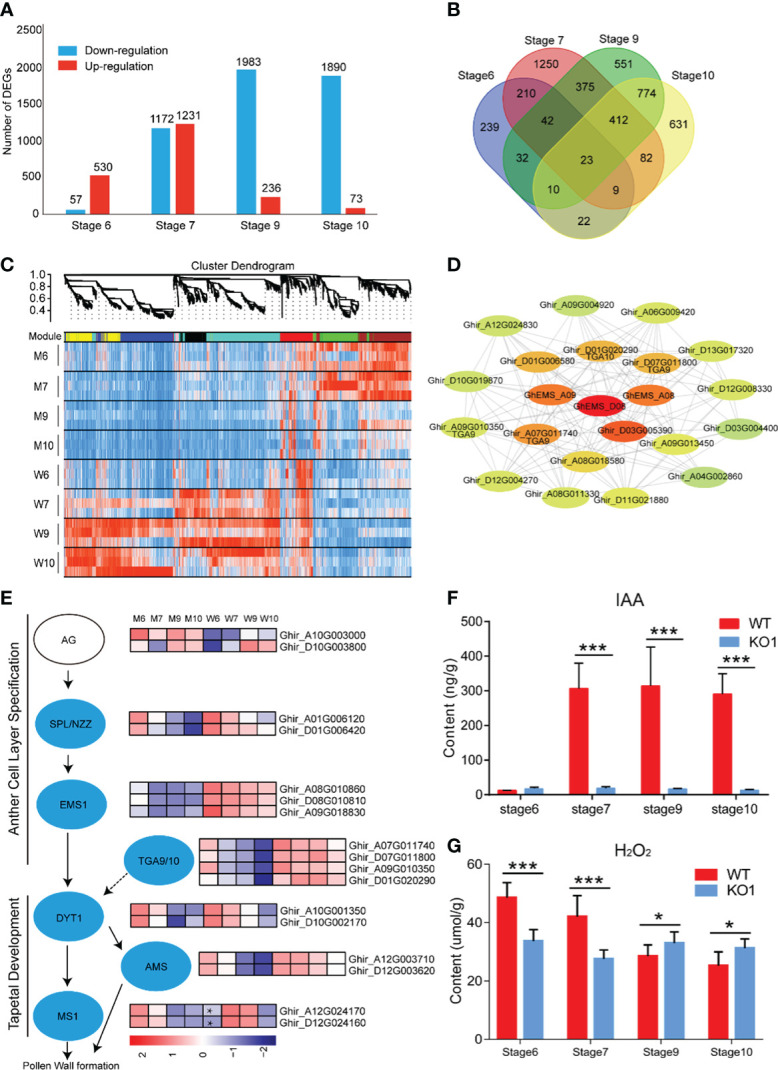
Transcriptome analysis of four developmental stages. **(A)** Difference gene statistics of wild type and mutant in four periods; **(B)** Intersection of differential genes at different stages; **(C)** WGCNA co-expression module, a total of 8 different modules; **(D)** Analysis of the co-expression network of the red module. The shades of color represent correlations; **(E)** Gene expression of the regulatory network of pollen development; **(F)** IAA content at four developmental stages; **(G)** H_2_O_2_ content at four developmental stages. W, wild type; M, mutant; *AG*, *AGAMOUS*; *AMS*, *ABORTED MICROSPORE*; *DYT1*, *DYSFUNCTIONAL TAPETUM 1*; *EMS1*, *EXCESS MICROSPOROCYTES 1*; *MS1*, *MALE STERILITY 1*; *NZZ/SPL*, *SPOROCYTELESS/NOZZLE*. Data are presented as means ± SE from five biologically independent experiments. Asterisks indicate statistically significant differences (***, *P <*0.001; *, *P <*0.05); by Student’s *t*-test.

To explore the molecular mechanisms of anther abortion in *GhEMS1* mutants, we compared the transcriptomes of WT and *Ghems1* anthers at four developmental stages (stages 6, 7, 9, 10). A total of 7,172 genes were found to be differentially expressed between *Ghems1* and WT at four anther developmental stages (**Supplementary Table 4**). Of these differentially expressed genes (DEGs), 2,070 (28.86%) were up-regulated and 5,102 (71.14%) were down-regulated (|log_2_(fold change)|≥1 and padj < 0.05) in *Ghems1* (**Figure 6A**). Compared with WT, *Ghems1* had more up-regulated genes than down-regulated genes at stages 6 and 7 (**Figure 6A**). However, at stages 9 and 10, *Ghems1* had more down-regulated genes than up-regulated genes (**Figure 6A**). Of these genes, 239, 1,250, 551 and 631 genes were unique at stages 6, 7, 9 and 10, respectively, and 23 genes showed differential expression in all four developmental stages (**Supplementary Table 5** and **Figure 6B**). These 23 genes included three *GhEMS1* genes that have been edited, and the expression of three *GhEMS1* genes was downregulated in all four stage mutant anthers (**Figure 6E**). The expression level of *GhEMS_D08* was higher than that of *GhEMS_A08/A09*, and gradually decreased following the anther development. GO enrichment analysis, “peroxidase activity” was highly enriched in stage 6 and 7 anthers of mutants, and “monooxygenase activity” is highly enriched in stage 6, 7, 9, which is related to peroxide in GO enrichment analysis (**Supplementary Figure 4** and **Table 6**). So, we detected the content of peroxide at four developmental stages of *Ghems1* and WT anthers (**Figure 6G**). The results showed that compared with the WT, the mutants had lower peroxide content in stage 6 and 7, but higher peroxide content in stage 9 and 10. Black spots began to appear in stage 7 and appeared in large numbers in stage 9, might be responsible for the appearance of black spots on the surface of anthers. In mutant stage 6 down-regulated genes, “activation of MAPKK activity” and “MAP kinase kinase kinase activity” were enriched. In stage 6, 9 down-regulated genes, “pollen exine formation” was enriched, while in stage 9 and 10 DEGs was enriched in “plant- type cell wall modification”, possibly related to pollen formation (**Supplementary Figure 4** and **Table 6**).

To generate co-expression networks for all DEGs and biological samples, the weighted gene co-expression network analysis was performed. A total of 8 gene modules were identified (**Figure 6C**). *EMS1* genes were distributed in the red module. We extracted the red module genes that may be related to *GhEMS1* genes to do a further co-expression network analysis and showed that there are four leucine-zipper transcription factors *TGACG9/10* (*TGA9/10*, *Ghir_D01G020290*, *Ghir_A07G011740*, *Ghir_D07G011800* and *Ghir_A09G010350*) genes, one tetrapeptide alpha-pyrone reductase 1 (*TKPR1*, *Ghir_D03G005390*), and one indole-3-acetic acid-amido synthetase (*GH3.6*, *Ghir_D01G006580*), were associated with *GhEMS1*. *TGA9* and *TGA10* are expressed throughout early anther primordia, and mutations in *TGA9* and *TGA10* lead to male sterility and differential defects in abaxial (Murmu et al., 2010). In the genetic framework for control of anthers development (Wilson and Zhang, 2009), the expression trends of *SPL/NZZ*, *EMS1*, *TGA9/10*, *DYT1* and *AMS* were the same, but *MS1* in the stage 6 anthers of mutant was higher than WT (**Figure 6E**). Previously reported that *TGA9/10* was located downstream of *SPL/NZZ* and upstream or in parallel with *DYT1* in the genetic hierarchy that controls anther development (Murmu et al., 2010), similar to *EMS1*. Interestingly, we also found that there was co-expression of *TGA9/10* and *EMS1* (**Figure 6D**), it was suggested that *TGA9/10* and *EMS1* may interact with each other to regulate anther development.

In the co-expression network, we found *TKPR1* which co-expressed with *EMS1* (**Figure 6D**), *TKPR1* involved in the biosynthesis of hydroxylated tetraketide compounds that serve as sporopollenin precursors (the main constituents of exine) was essential for pollen wall development (Tang et al., 2009; Grienenberger et al., 2010). And GO enrichment analysis shows that “pollen exine formation” was highly enriched in stage 6 and 9 anthers of mutants. To confirm whether the synthesis of sporopollenin precursors in the mutants had been affected, we measured the autofluorescence intensity of sporopollenin of *Ghems1* and WT anthers at stages 6, 7, 9, 10 by microscopy with UV light illumination (**Supplementary Figure 5**) (Ma et al., 2022). In the mutant, the autofluorescence of sporopollenin was not detected in the four stages, but in WT, the autofluorescence of sporopollenin was detected in the 9 and 10 stages. This showed that the synthesis of sporopollenin was affected in the mutant.

Phytohormones play an important role in the regulation of anther development. In the co-expression network, we found *GH3.6* which co-expressed with *EMS1* (**Figure 6D**), *GH3.6* catalyzes the synthesis of indole-3-acetic acid (IAA)-amino acid conjugates, providing a mechanism for the plant to cope with the presence of excess auxin (Staswick et al., 2005). Compared with WT, the expression of *GH3.6* in the mutant gradually decreased (**Supplementary Figure 6**). So, we detected the dynamic changes of auxin (IAA) in anthers of *Ghems1s* male sterile line (**Figure 6F**). During the four anther developmental stages, the free IAA content of male sterile plants *KO1* changed little with the development of anther and was at a lower level. However, the free IAA content of WT increased greatly between the stage 6 and stage 7 of anthers development, and maintained a high level after that. The lower IAA content in the stage 7, 9, 10 anthers of *Ghems1s* mutants may be closely related to the occurrence of male sterility.

From the above results, we found that after the editing of both *GhEMS1_A08* and *GhEMS1_D08*, there were yellow spots on the surface of the anthers and a few fertile pollen grains in the chamber. Moreover, the results of simultaneously editing three *GhEMS1* genes showed that there were necrosis-like dark spots on the surface of the anthers, which contained completely aborted pollen grains, thus the necrosis-like dark spots can serve as a marker of completely male-sterile cotton lines with *GhEMS1s* mutants, and can help breeders to screen sterile plants at the early anther stage.

## Discussion

In recent years, CRISPR/Cas9 technology has played an important role in the creation of sterile materials. The sterile genes cloned in model plants *Arabidopsis* and rice have potential applications in cotton. *AtEMS1*, *OsMSP1* encode LRR receptor kinases, and the mutants have the same phenotype, including the production of a large number of microspore mother cells, no tapetum layer and middle layer. Compared with *Arabidopsis thaliana*, cotton *GhEMS1* mutant anther surface has obvious necrotic phenotype, and the molecular mechanism needs to be further studied.

Previous reports have shown that the young microspore stage and flowering stage in rice are very sensitive to high temperature (HT) stress, and HT stress destroys the function of tapetum during microspore formation and leads to poor anther dehiscence (Endo et al., 2009). It was worth noting that the expression of *GhEMS1* genes at the tetrad stage were differently respond to HT in the HT-tolerant and -sensitive lines, could provide a theoretical basis for the study of male sterility of cotton caused by high temperature (**Supplementary Figure 7**).

Morphological trait markers have broad application prospects in cotton production. Among them, pigment glands, okra leaf shape, and virescently traits were more commonly studied (Zhu et al., 2008; Ma et al., 2013; Ma et al., 2016). The virescently marker is linked to sterility gene and can be used to identify sterile lines at the early seedling stage (Ma et al., 2013). For now, there has been no marker found on the anther associated with fertility. In our study, the necrosis-like dark spots appeared on the anthers in the early stage, which could be screened during early bud periods, reducing waste of resources and allowing for hybrid breeding.

Upland cotton is a polyploid species with a larger genome (2.5 Gb), so most genes have multiple copies and high sequence similarity due to the polyploidization of *At* and *Dt* sub-genomes, which makes cotton gene engineering very difficult (Wang et al., 2019). In this study, many *EMS1*-like genes were aligned in the upland cotton genome through the amino acid sequence of the *Arabidopsis AtEMS1* gene. The three genes of *GhEMS1_A08, GhEMS1_D08* and *GhEMS1_A09* may have functional redundancy because they are in one branch and the mutant is completely male sterile by knocking out *GhEMS1_A08, GhEMS1_D08* and *GhEMS1_A09* at the same time, but the two gene mutants show partial infertility by knocking out *GhEMS1_A08, GhEMS1_D08.* Multiple genes control male sterility, which makes it difficult to find *EMS* genes using map-based cloning. Completely sterile plants cannot produce tapetum and intermediate layers, confirming that cotton *GhEMS1* is a key gene regulating cotton anther and microspore development. Thus, to establish a cotton hybrid system, the *GhEMS1* mutant can be used as the male sterile line, and the negative sterile plants (Cas9-free) can be selected and crossed with other cultivars to create excellent hybrids. The positive sterile plants (with Cas9) can be crossed with transgenic acceptors or cultivars to breed sterile lines (**Supplementary Figure 8**).

The effect of IAA on plant male sterility was often reported. The reduction of the express of IAA related gene in wheat was associated with the occurrence of male-sterility (Su et al., 2019). At the third stage, the contents of IAA, GA_3_ and ZR in CMS were remarkably lower than its maintainer of Chinese Cabbage (Liu et al., 2014). When IAA was depleted, the vascular bundles develop abnormally, and the passage of water and nutrients into the drug compartment was blocked, resulting in abnormal microspore development and pollen abortion (Liu et al., 2014). ABA and IAA are involved in PCD of microsporocytes during meiosis in *Petunia hybrida* L. (Kovaleva et al., 2018). Sugar and IAA may be the key regulators of cotton anther response to high temperature stress (Min et al., 2014). In this study, we found that among the genes co-expressed by *EMS1* through the co-expression network, there was an auxin synthesis related gene *GH3.6*. At the same time, there was a significant difference in IAA content between the male sterile mutant and the wild type during the 6-10 stage of anthers development, which is very likely to be the cause of male sterility. We also tested other endogenous hormones, but found no regular changes. As to whether the male sterility can be restored by spraying IAA, further study is needed.

In summary, we created a new male-sterile cotton line, which may further promote the utilization of the genic male-sterile lines and the development of cotton hybrid breeding. In addition, the cotton line with three mutated *EMS1* homolog genes provides the basis for the study of cotton anther tapetum and microspore development.

## Data availability statement

The original contributions presented in the study are publicly available. This data can be found here: NCBI, PRJNA827503.

## Author contributions

XG, XZ, LM and JZ designed the studies. JZ, XX, NL, PW, SW, YZ, XW, SC, YM performed the experiments and data analysis. WL, and HM improved the grammar of the manuscript. JZ, PW, XG and LM wrote the manuscript. All authors contributed to the article and approved the submitted version.
