# Supplementary material for: A male-sterile mutant with necrosis-like dark spots on anthers was generated in cotton
Source: Front Plant Sci. 2023 Jan 9;13:1102196. doi: 10.3389/fpls.2022.1102196 (PMC9868585; doi:10.3389/fpls.2022.1102196)
Supplement: Supplementary file 1 [file DataSheet_1.docx]

**Supplementary Information for**

**A male-sterile mutant with necrosis-like dark spots on anthers was generated in cotton**

Jun Zhang^1,2†^, Peng Wu^1†^, Ning Li^1†^, Xiaolan Xu^1^, Songxin Wang^1^, Siyuan Chang^1^, Yuping Zhang^1^, Xingxing Wang^1^, Wangshu Liu^2^, Yizan Ma^1^, Hakim Manghwar^1,3^, Ling Min^1*^, Xiaoping Guo^1*^

^1^ National Key Laboratory of Crop Genetic Improvement & Hubei Hongshan Laboratory, Huazhong Agricultural University, Wuhan, China.

^2^ Zhejiang Provincial Key Laboratory of Crop Genetic Resources, Institute of Crop Science, Plant Precision Breeding Academy, College of Agriculture and Biotechnology, Zhejiang University, Hangzhou, China.

^3^ State Key Laboratory for Conservation and Utilization of Subtropical Agro-Bioresources, South China Agricultural University, 510642 Guangzhou, China.

† These authors contributed equally to this work.

* Corresponding author: Xiaoping Guo (xpguo@mail.hzau.edu.cn) and Ling Min (lingmin@mail.hzau.edu.cn)

**SUPPORTING INFORMATION**

**Supplementary Figure 1. EMS1 amino acid sequences alignment.**

**Supplementary Figure 2. The Agrobacterium-mediated genetic transformation and plant regeneration of transgenic plants**.

**Supplementary Figure 3. T0 mutant’s gene editing detection**

**Supplementary Figure 4. GO enrichment of differential genes.**

**Supplementary Figure 5. Sporopollenin autofluorescence in KO1 and WT.**

**Supplementary Figure 6. Differential expression of *GH3.6* and *TKPR1*.**

**Supplementary Figure 7. Expression of three *GhEMS1* genes at the tetrad stage (6- to 7-mm bud) in *G. hirsutum* 84021 (HT tolerant) and H05 (HT sensitive) under the two conditions determined by transcriptome sequencing.**

**Supplementary Figure 8. Hybrid breeding system**.

**
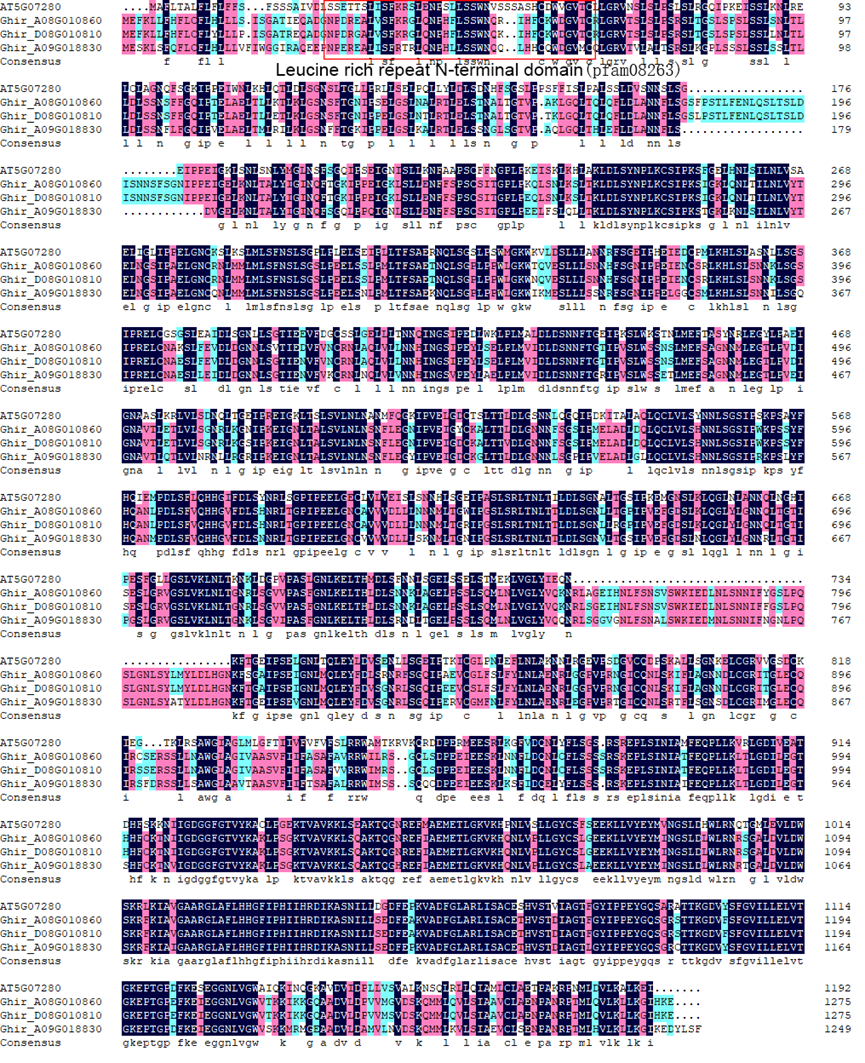
**

**Supplementary Figure 1. EMS1 amino acid sequences alignment.** *Ghir_A08G010860*, *Ghir_D08G010810*, *Ghir_A09G018830*, and *AT5G07280* have leucine rich repeat N-terminal domain and leucine-rich repeat sequences. *AT5G07280* encodes *EXCESS MICROSPOROCYTES1*.


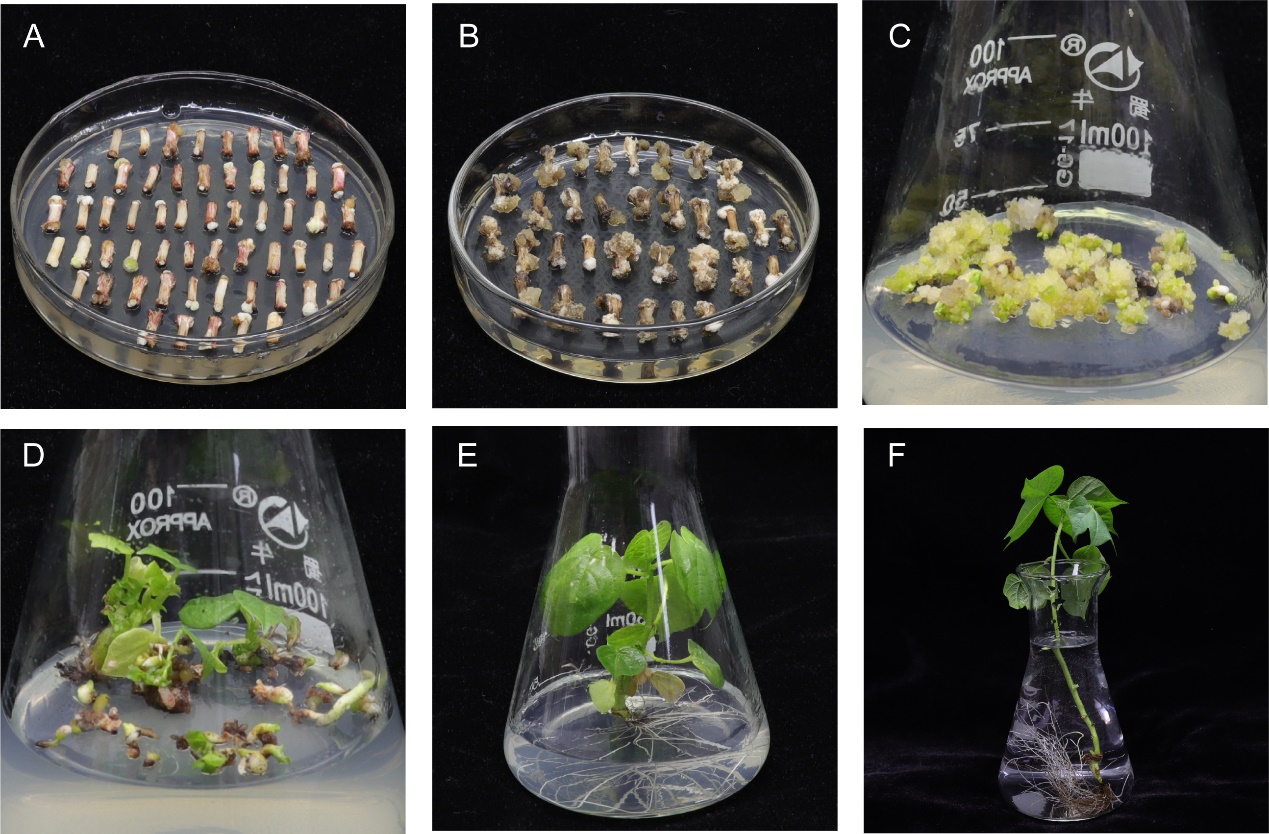


**Supplementary Figure 2. The Agrobacterium-mediated genetic transformation and plant regeneration of transgenic plants**. (**A**-**B**) Callus induction. (**C**) Somatic embryogenesis. (**D**-**F**) Plant regeneration.


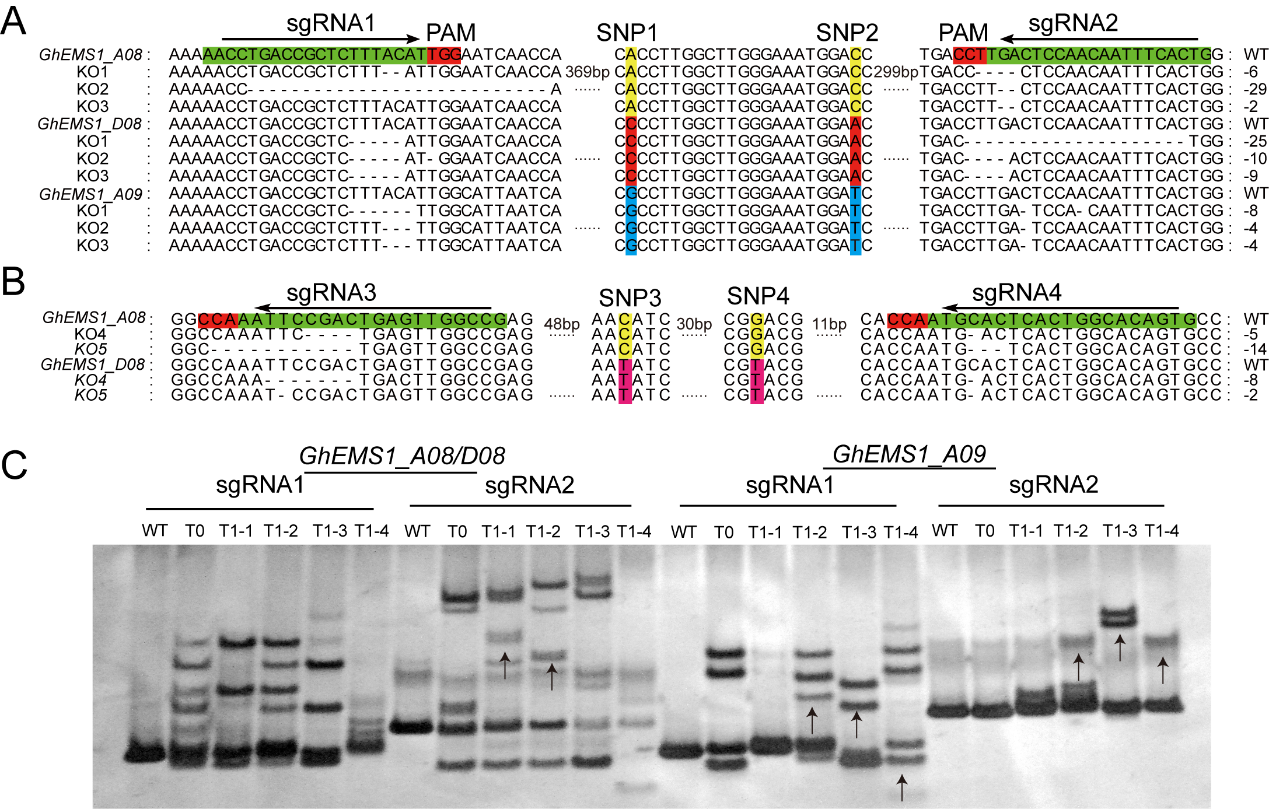


**Supplementary Figure 3. T0 mutant’s gene editing detection**

(**A**-**B**) Detection of the editing of target sites in five transgenic plants. The sgRNA target sites and the PAM regions are highlighted in green and red backgrounds, respectively. The length of the deletion is shown on the right. The gaps between the omitted nucleotides are indicated by dotted lines, and their lengths are labeled above the lines. (**C**) Detection inheritance of gene editing in target genes using PAGE method. The sgRNA sites of *GhEMS1_A08/D08* were simultaneously amplified by PCR due to their similar sequences, and two sgRNA sites of *GhEMS1_A09* were amplified separately. WT was used as a negative control, T0 generation (KO1) and T1 generation plants were selected for display. New edit types indicated with black arrow. PAM, protospacer adjacent motif; PAGE, polyacrylamide gel electrophoresis.


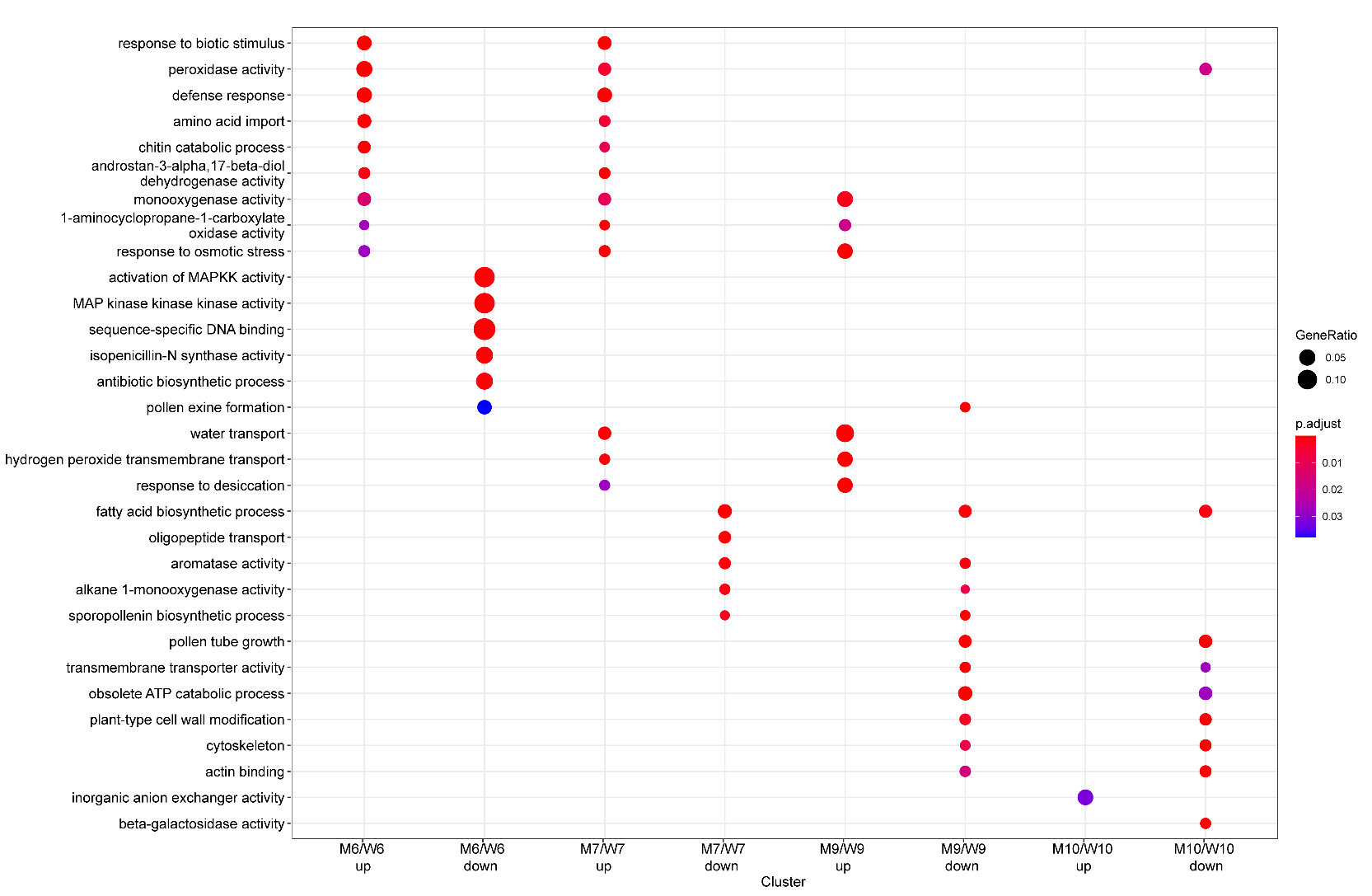


**Supplementary Figure 4.** **GO enrichment of differential genes.**

W6~W10, WT anthers at stages 6, 7, 9, 10; M6~M10, *Ghems1* anthers at stages 6, 7, 9, 10

**
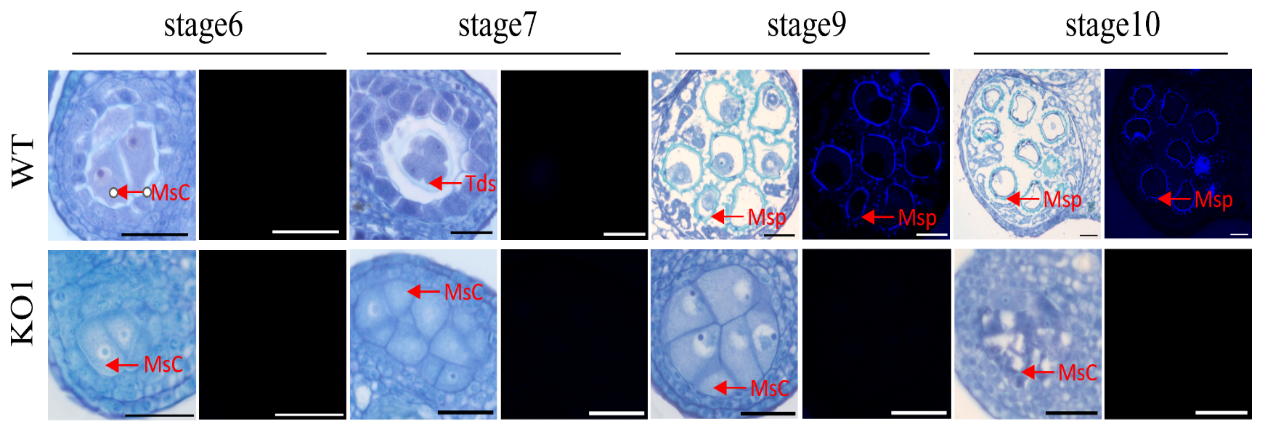
Supplementary Figure 5.** **Sporopollenin autofluorescence in KO1 and WT.** Msc, microsporocyte; Tds, tetrads; Msp, microspore; WT, wild type. KO1, GhEMS 3 genes simultaneous mutant. Scale bars: 20 µm.


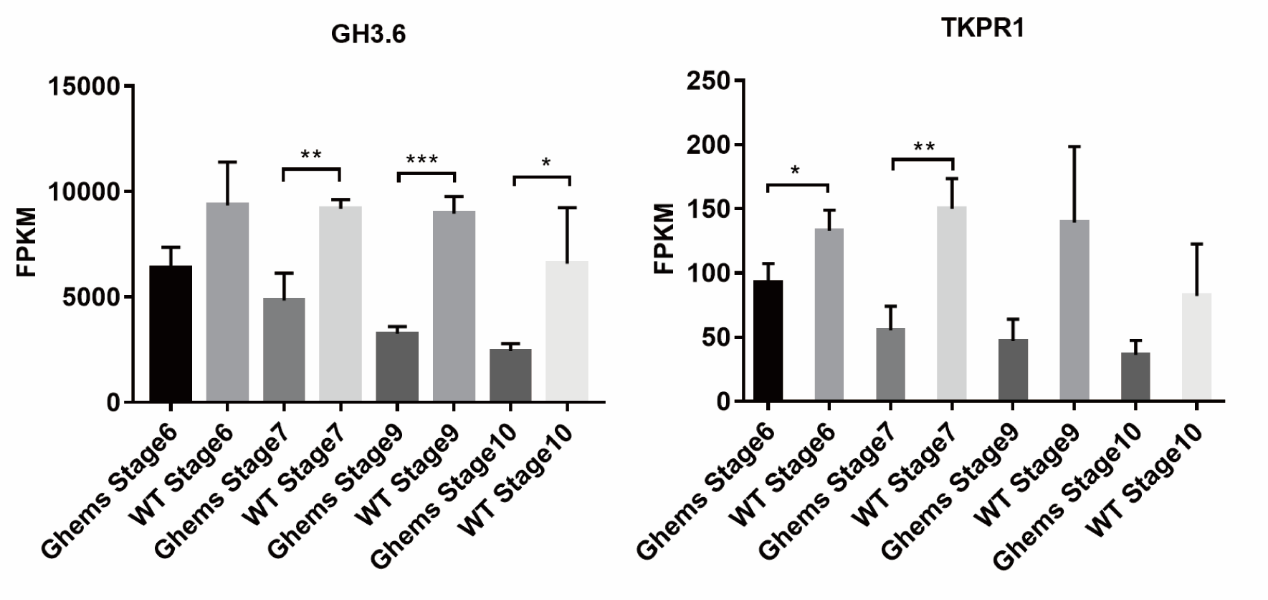


**Supplementary Figure 6.** **Differential expression of *GH3.6* and *TKPR1***


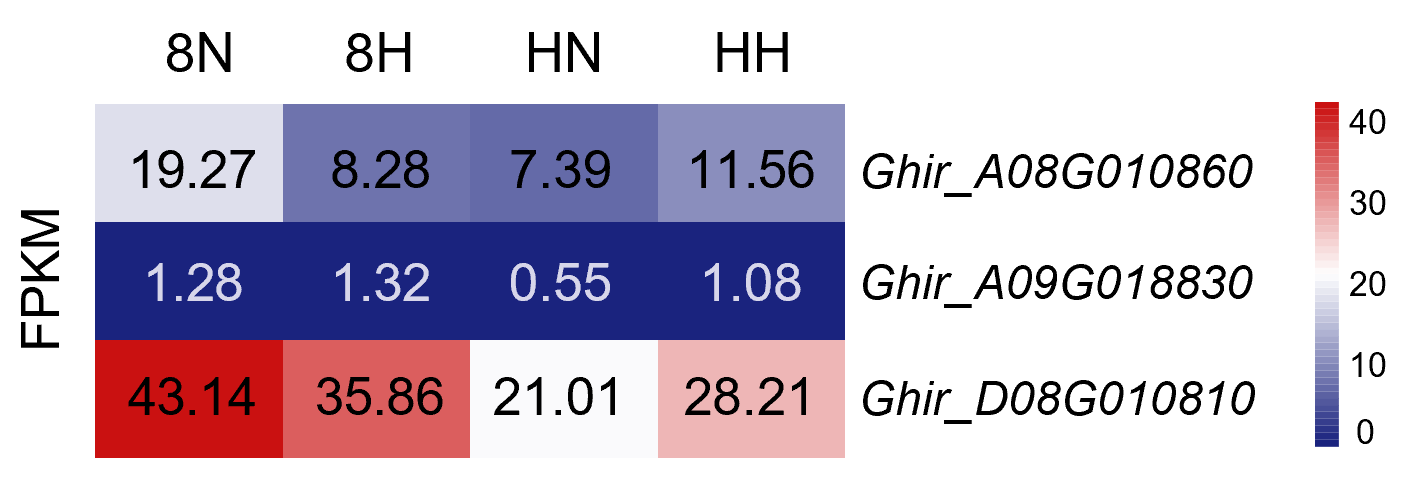


**Supplementary Figure 7. Expression of three *GhEMS1* genes at the tetrad stage (6- to 7-mm bud) in *G. hirsutum* 84021 (HT tolerant) and H05 (HT sensitive) under the two conditions determined by transcriptome sequencing.** 8N and 8H refer to 84021 under normal temperature and high temperature conditions, respectively; HN and HH refer to H05 under normal temperature and high temperature conditions. FPKM，Fragments Per Kilobase of exon model per Million mapped fragments.


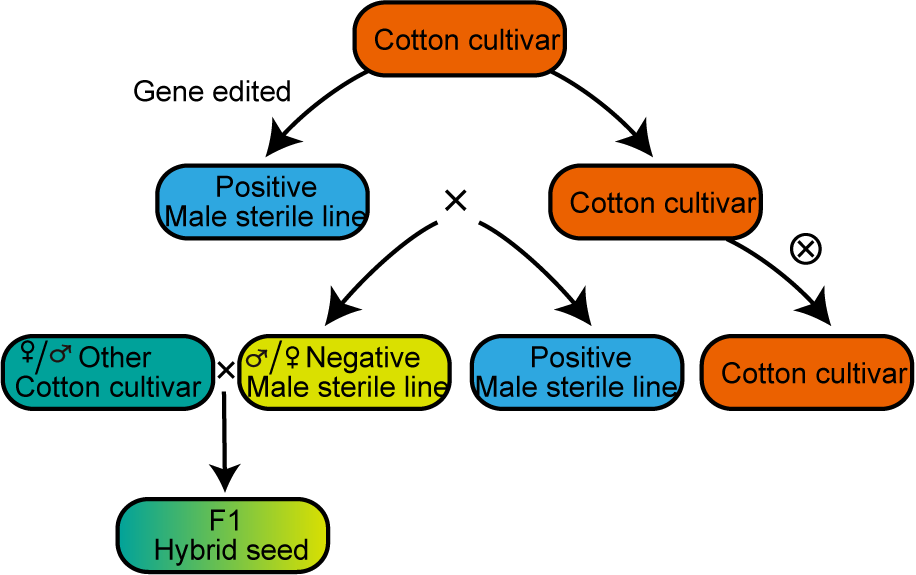


**Supplementary Figure 8. Hybrid breeding system**. Editing cotton cultivars to create sterile lines, and the Cas9-free sterile plants can be selected and crossed with other cultivars to create excellent hybrids. The positive sterile plants (with Cas9) can be crossed with transgenic acceptors or cultivars to breed sterile lines.
